# Cost-Effectiveness of Low-Field Intraoperative Magnetic Resonance in Glioma Surgery

**DOI:** 10.3389/fonc.2020.586679

**Published:** 2020-11-02

**Authors:** Sergio Garcia-Garcia, Borja García-Lorenzo, Pedro Roldan Ramos, Jose Juan Gonzalez-Sanchez, Diego Culebras, Gabriela Restovic, Estanis Alcover, Imma Pons, Jorge Torales, Luis Reyes, Laura Sampietro-Colom, Joaquim Enseñat

**Affiliations:** ^1^ Department of Neurological Surgery, Hospital Clinic, Barcelona, Spain; ^2^ Assessment of Innovations and New Technologies Unit, Hospital Clínic, University of Barcelona, Barcelona, Spain; ^3^ Economic and Financial Management Department, Hospital Clinic, Barcelona, Spain

**Keywords:** cost-effectiveness, glioma, incremental cost-effective ratio, intraoperative magnetic resonance, technology assessment, Karnofsky performance status, progression-free survival (PFS)

## Abstract

**Object:**

Low-field intraoperative magnetic resonance (LF-iMR) has demonstrated a slight increase in the extent of resection of intra-axial tumors while preserving patient`s neurological outcomes. However, whether this improvement is cost-effective or not is still matter of controversy. In this clinical investigation we sought to evaluate the cost-effectiveness of the implementation of a LF-iMR in glioma surgery.

**Methods:**

Patients undergoing LF-iMR guided glioma surgery with gross total resection (GTR) intention were prospectively collected and compared to an historical cohort operated without this technology. Socio-demographic and clinical variables (pre and postoperative KPS; histopathological classification; Extent of resection; postoperative complications; need of re-intervention within the first year and 1-year postoperative survival) were collected and analyzed. Effectiveness variables were assessed in both groups: Postoperative Karnofsky performance status scale (pKPS); overall survival (OS); Progression-free survival (PFS); and a variable accounting for the number of patients with a greater than subtotal resection and same or higher postoperative KPS (R-KPS). All preoperative, procedural and postoperative costs linked to the treatment were considered for the cost-effectiveness analysis (diagnostic procedures, prosthesis, operating time, hospitalization, consumables, LF-iMR device, etc). Deterministic and probabilistic simulations were conducted to evaluate the consistency of our analysis.

**Results:**

50 patients were operated with LF-iMR assistance, while 146 belonged to the control group. GTR rate, pKPS, R-KPS, PFS, and 1-year OS were respectively 13,8% (not significative), 7 points (p < 0.05), 17% (p < 0.05), 38 days (p < 0.05), and 3.7% (not significative) higher in the intervention group. Cost-effectiveness analysis showed a mean incremental cost per patient of 789 € in the intervention group. Incremental cost-effectiveness ratios were 111 € per additional point of pKPS, 21 € per additional day free of progression, and 46 € per additional percentage point of R-KPS.

**Conclusion:**

Glioma patients operated under LF-iMR guidance experience a better functional outcome, higher resection rates, less complications, better PFS rates but similar life expectancy compared to conventional techniques. In terms of efficiency, LF-iMR is very close to be a dominant technology in terms of R-KPS, PFS and pKPS.

## Introduction

Primary brain tumors may manifest with a wide range of signs and symptoms such as seizures, headache, neurological deficits, and endocrinological disturbances, alone or in combination. The most frequent and malignant primary brain tumor in adults is the Glioblastoma (GBM), a subtype within the group of high-grade gliomas (HGG) (1). The therapeutic algorithm for these malignant neoplasms consists of a combination of surgery, chemotherapy, and radiotherapy, together with some new emergent treatments (2). Besides other prognostic factors such as Karnofsky performance status (KPS), histology, and molecular markers; the extent of resection (EoR) is a major prognostic factor for overall survival (OS) and progression-free survival (PFS) in this subset of malignant gliomas (3, 4). Recently, some authors have reported the benefits of supramarginal resection in the clinical outcome of HGG patients (5). Moreover, during the last decade, substantial evidence has been accrued to support the relevance of maximal or supramarginal resection in low-grade gliomas (LGG) (6, 7). In this sense, several studies have proved the role of surgery in the control of seizures, OS, and PFS for LGG (6, 8, 9). All in all, EoR plays a major role in the treatment and clinical course of patients diagnosed with either grade of glioma.

Therefore, maximal safe resection is a major goal in LGG and HGG treatment. Over the last decades neurosurgeons have considerably improved their outcomes pushing the boundaries of which had been considered feasible. These advancements have been supported by a significant technological development. Regarding glioma surgery the main innovations occurred in the field of intraoperative image where intraoperative magnetic resonance (iMR); ultrasonography and 5-ALA fluorescence guided surgeries gained momentum becoming a sign of excellence (10). Specifically, iMR guided surgery has consistently demonstrated an increment in the rates of gross total resection (GTR) without a negative impact in neurological outcomes. This being said, the evidence that links this rise in resection rates with an improvement in OS and PFS is poor or, in other words, indirect (3, 10, 11).

Consequently, the general adoption of these devices is supported by low quality evidence. Moreover, cost analysis of the effectiveness of iMR in glioma surgery has not been thoroughly conducted. Economic evaluation consists of the comparison of costs and health benefits of alternative interventions. Its ultimate goal is to provide information for decision makers who, in a scarce resource context, ought to make justified investment decisions based on the health improvements that are obtained. Novel technologies often yield better health outcomes entailing higher costs. The economic evaluation, through cost-effectiveness analyses, estimate the incremental cost per unit of health effectiveness gained by a given technology. Although the economic impact of the current technological escalade is often disregarded by neurosurgeons, it actually is a paramount issue that should be addressed to maintain surgical excellence. Nonetheless, nowadays, there are not scientifically proven advantages, neither in terms of cost savings, clinical efficacy nor in cost-effectiveness to establish iMR as a state-of-the-art asset in glioma surgery.

In this clinical research, we sought to assess the economic impact of the implementation of a low field iMR (LF-iMR) device in glioma surgery. Then, we compare the cost and clinical outcomes of two different cohorts of patients, one treated with LF-iMR assistance and another one operated in a conventional fashion.

## Methods

The present clinical investigation was designed as a hybrid study in which a prospectively recruited cohort was compared to an historic cohort. The latter (control cohort) was made up with consecutive patients treated from LGG (Pilocytic Astrocytoma, Diffuse astrocytoma, Oligodendroglioma, Pleomorphic Xanthoastrocytoma, Chordoid Glioma, and Angiocentric glioma), HGG (Glioblastoma, Anaplastic astrocytoma, Anaplastic oligodendroglioma, and Gliosarcoma) with a maximal safe resection intention between 2010 and 2013 at our institution. The prospective (intervention) cohort consisted of patients operated of LGG and HGG with a maximal safe resection intention with the assistance of a LF-iMR (PoleStar N-20, Odin Medical Technologies, Yokneam, Israel and Medtronic, Louisville, CO, USA). These patients were recruited between June 2013, date of the installation of the device, and June 2016. Tumors near eloquent areas were not excluded in either cohort.

The present research was approved by the institutional review board (HCB/2013/8782). Patients signed an informed consent before surgery agreeing the use of the LF-iMR; the review and analysis of their clinical records; and the publication of the results derived from this research.

The present report adheres to the recommendations of the ISPOR Task Force and the guidelines contained in the document named *Consolidated Health Economic Evaluation Reporting Standards* (CHEERS) for economic evaluations of health interventions (12).

### Surgical Technique

The retrospective group was operated using conventional microsurgery, which could include the use of an ultrasonic aspirator and standard neuronavigation. Patients operated with any other intraoperative image assistance or intraoperative fluorescence were excluded. The surgical workflow for the LF-iMR group has been previously described (13–15). Intraoperative neurophysiologic monitoring was implemented in both cohorts, according to the decision of the surgical team. Neurophysiologic criteria for stopping the resection remained unchanged across the time of the present study.

### Extent of Resection

EoR was defined under the following thresholds: GTR if 100% of the mass was removed; Subtotal resection if more than 90% of the mass was removed; anything else under this value was considered a partial resection (PR) or a biopsy. In HGG, the mass volume considered to evaluate the EoR was the corresponding to the contrast enhancing part of the lesion. In LGG, the whole T2/FLAIR hyperintense volume was considered.

### Cost-Effectiveness

A cost-effectiveness analysis comparing conventional microsurgery and LF-iMR guided surgery was conducted. Socio-demographic (age and gender) and clinical variables [pre and postoperative KPS (pKPS), histopathological classification, EoR; postoperative complications, need of re-intervention within the first year, 1-year progression-free survival (PFS), and 1-year postoperative survival] were analyzed in both groups. One-year PFS was computed as the time in days from surgery to the first radiological exam demonstrating progression or recurrence within the first year of follow-up. Patients with progressions or recurrences later than one year were censored at 365 days. In those patients in which resections were partial, recurrence or progressions were considered when the tumor demonstrated growth or novel malignant features compared to the residual postoperative tumor. Postoperative complications included local (intraparenchymal haemorrhage, wound, and surgical site infection), neurological (new neurological deficits, new onset seizures, hydrocephalus, ischemic stroke, and symptomatic cerebral oedema), and systemic (cardiac infarction, deep venous thrombosis, and pulmonary thromboembolism) complications. Patients were followed-up for a minimum of 2 years after surgery.

We extracted the resource use of every single patient from the hospital administrative database, and then we associated the corresponding unit cost to each resource use in order to compute the cost per individual patient. The collected resource use were: operating time (in minutes); hospitalization in the intensive care unit in days (ICU) which includes wages of involved professionals and consumables (meals, medicines, etc.); prosthesis (which includes dural substitutes, miniplates, sterile covers and drapes, etc.); cost of the LF-iMR device; type and number of radiological images performed one year after the intervention and length of stay (LoS) in days (see **Table 1**). The cost in Euros-2018 of these resources was pooled for further analysis. No discount rate was considered due to the short time frame of the analysis (16).

**Table 1:** Resource use and unit costs.

	Resource use		Probability distribution^a^	Unit costs^b^
	Conventional OR	LF-iMR		
**OR**				
Time in minutes [mean (SD)]	368 (75)	415 (70)	Gamma	5 €
Surgical pack [% (n)]	100 (146)	100 (50)	n.v.	1,150 €
Navigation system [%o(n)]	0 (0)	100 (50)	n.v.	862 €
Prosthesis [% (n)]	92 (134)	88 (44)	Beta	272 €
LF-iMR [% (n)]	0 (0)	100 (50)	n.v.	1M €^c^ 833 €^d^
**ICU**				
% (n)	67 (98)	34 (17)	Beta	555 €
**Preoperative images [mean (SD)]**				
MR-C	1.2 (0.9)	1.0 (0.9)	Gamma	203 €
MR	2.0 (1.63)	1.7 (1.7)		138 €
PET	0.1 (0.2)	0.1 (0.3)		566 €
X-Ray	1.3 (1.6)	1.1 (1.2)		15 €
Portable XRay	0.03 (0.2)	0.1 (0.5)		32 €
CT	0.5 (0.6)	0.4 (0.6)		72 €
**Postoperative images [mean (SD)]**				
MR-C	3.0 (1.7)	2.5 (1.7)	Gamma	203 €
MR	3.5 (1.8)	3.1 (1.6)		138 €
X-Ray	1.1 (2.9)	1.7 (3.5)		15 €
Portable XRay	0.9 (1.2)	0.3 (0.9)		32 €
SPECT	0.1 (0.1)	0.1 (0.3)		166 €
CT	1.1 (2.9)	0.7 (1.4)		72 €
**Hospitalization**				
Length of stay in days [mean (SD)]	11.9 (7.8)	9.7 (5.4)	Gamma	422 €

ICU, Intensive care unit hospitalization; MR-C, magnetic resonance with contrast; M, Million; n.v., not varied; OR, operating room.

^a^Probability distribution of every resource use parameter.

^b^All cost parameters were varied using the Gamma distribution.

^c^Total cost of the iMR device.

^d^Cost per intervention using iMR based on the life cycle (10 years) and the potential number of patients (n = 120) who yearly benefit from the iMR device.

In order to define the cost of LF-iMR imputed to each patient we did not only consider the surgeries object of the current analysis. Conversely, we inferred the cost per patient from all the indications in which we currently use the LF-iMR: intrinsic and extrinsic brain lesions, cavernomas, pituitary macroadenomas and epilepsy surgery. Thus, the cost of the device per patient was calculated considering a life cycle of 10 years and a potential number of 120 surgeries per year [50 oncological surgeries (13, 15), 30 pituitary macroadenomas resection (14), 20 cavernous malformations resections, 20 epilepsy surgeries] (**Table 1**).

A descriptive analysis of socio-demographic, clinical, and resource usage variables was conducted. These variables were compared by a t-test to discard significant differences between groups. Patient’s specific data were pooled to compare effectiveness, costs, and outcomes between groups. As effectiveness variables mean postoperative KPS, 1-year OS and 1-year PFS were considered. In addition, a combined clinical variable named R-KPS was created to assess effectiveness. This dichotomic variable was used to classify patients onto two categories, one for those with total or subtotal resections and conserved or improved KPS and another one for those with whichever degree of resection and a worsening in the KPS. The proportion of patients with total or subtotal resections and conserved or improved KPS was considered as an additional effectiveness variable.

Costs were computted as the mean costs per patient. Mean incremental effectiveness and costs were calculated for each surgical group. Mean incremental effectiveness was calculated as the difference between the mean effectiveness of the LF-iMR and the conventional microsurgery. The same operation was implemented to calculate mean incremental costs. Finally, the cost-effectiveness analysis was summarized as the incremental cost-effectiveness ratio (ICER), defined as the incremental cost divided by the incremental effectiveness of two studied alternatives (17). The cost-effectiveness analysis took a hospital perspective considering only direct health costs incurred by the hospital.

Deterministic and probabilistic sensitivity analyses (PSAs) were conducted to evaluate the robustness of the results of the cost-effectiveness analysis. The deterministic analysis compares the results (ICERs) of the base case with various hypothetic scenarios to test the consistency of our results. Three different scenarios were built in which the effectiveness and the cost parameters of the intervention group were shifted to the extreme values (mean ± standard deviation). Then, a new ICER was calculated for each scenario. Scenario A was created to simulate a conservative situation in which the worst possible clinical outcomes in the LF-iMR group would be obtained. Conversely, Scenario B was built to offer a favorable scenario assuming the best possible clinical outcomes of this new technology. In Scenario C, clinical variables remained unchanged while the results in terms of costs were taken to a propitious setting in which the cost of the LF-iMR was reduced by 20% of the average price. Finally, in Scenario D, a sub-group analysis was conducted exclusively considering the HGG cohort.

To characterize the uncertainty of the model, we undertook a PSA using Monte Carlo simulation. We applied probability distributions to each parameter depending on its nature according to the Economic Evaluation Guidelines (17–19). The PSA was graphically represented by means of the cost-effectiveness planes.

Calculations were performed using Microsoft Excel XP™. Level of significance was set at p < 0.05.

## Results

The sample (N = 196) demonstrated a slight male predominance (60.7%) and an average age of 54 years. The majority of tumors (69.4%) were classified under the label of HGG. Mean preoperative KPS was rather favorable (score = 85). No statistically significant differences in demographic nor in clinical variables were found between groups (**Table 2**).

**Table 2:** Socio-demographic and clinical variables.

		Conventional ORn = 146	LF-iMRn = 50	p	Total
Gender	Female (n, %)	(57, 39)	(20, 40)	*0.892*	(77, 39.3)
Age (years)	Average	54 +/− 15	53 +/− 15	*0.685*	54 +/− 15
	Range	21–88	21–82		21–88
Karnofsky pre-surgery	Average	84 +/− 9	87 +/− 8	*0.088*	85 +/− 9
	Range	70–100	70–100		70–100
Histopathology	LOW GRADE (n, %)	(41, 28.10)	(19, 38.00)	*0.189*	(60, 30.6)
	Pilocytic Astrocytoma	13, 31.7	4, 21.1		17, 28.3
	Diffuse astrocytoma	10, 24.4	3, 15.8		13, 21.6
	Oligodendroglioma	12, 29.3	10, 52.6		22, 36.6
	Pleomorphic Xanthoastrocytoma	3, 7.3	0, 0		3, 5.0
	Chordoid Glioma	1, 2.4	1, 5.3		2, 3.3
	Angiocentric glioma	2, 4.9	1, 5.3		3, 5.0
	HIGH GRADE (n, %)	(105, 71.90)	(31, 62.00)		(136, 69.4)
	Glioblastoma Multiforme	76, 72.4	24, 77.4		100, 76.9
	Anaplastic Astrocytoma	9, 8.6	1, 3.2		10, 7.7
	Anaplastic Oligodendroglioma	14, 13.3	4, 12.9		18, 13.8
	Gliosarcoma	4, 3.8	2, 6.5		6, 4.6
	Meduloblastoma	2, 1.9	0, 0		2, 1.5
Reintervention	Yes (n, %)	(0, 0)	(4, 8)	*0.283*	(4, 2)

NS, No statistically significant at 5%.

### Clinical Outcomes

**Figure 1:**
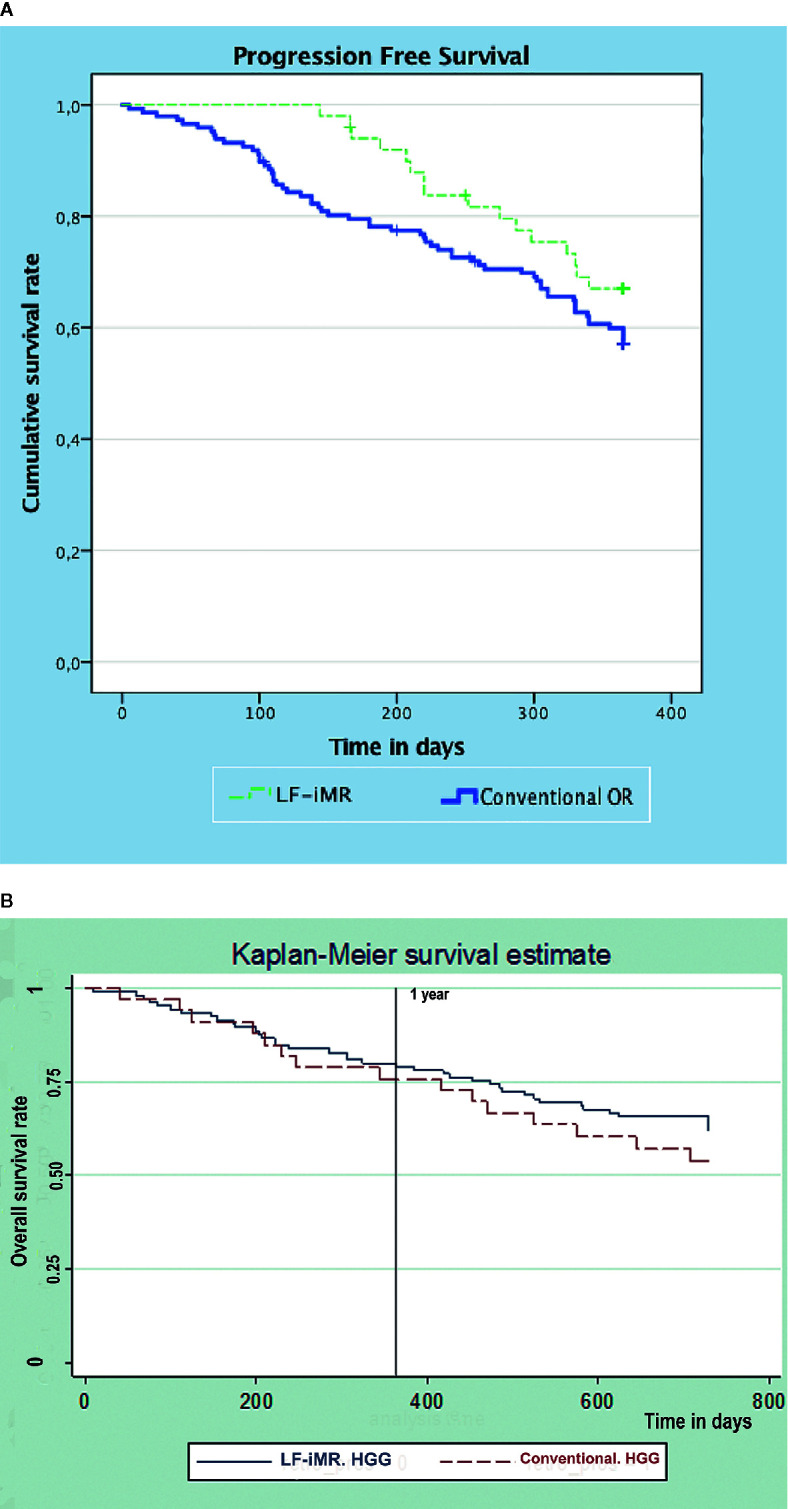
Kapplan-Meier survival curves. **(A)** Progression-free survival curves for LF-iMR and Conventional surgery cohorts. **(B)** Kapplan-Meier survival curves for high grade glioma patients.

The use of the LF-iMR, compared to conventional microsurgery, provided a higher percentage of patients with a GTR and a lower percentage of patients presenting with postoperative complications. Despite this positive trend related to the implementation of the LF-iMR, no statistically significant differences were found between groups regarding the EoR. Patients undergoing LF-iMR guided surgery presented a significant higher pKPS than those operated in a conventional fashion (84 vs.77; Standard Deviation (SD): 9; p < 0.000). One-year OS was higher for patients in the intervention group (88% vs. 85%; p > 0.05) while 2-years OS was lower (70% vs. 73.3%; p > 0.05). Regarding PFS a significant improvement was found in the LF-iMR cohort (333 days vs. 295 days; p = 0.025). A comparison between PFS distributions for both cohorts was conducted using the Log Rank or Mantel-Cox test obtaining a statistically significant result (p = 0.03) (**Figure 1A**). In addition, our study demonstrated the benefits of LF-iMR by means of the novel R-KPS effectiveness variable, since there was a significant increase in terms of number of patients in which a subtotal or GTR was obtained without worsening their pKPS in the intervention group (52% vs.35%; SD: 10.4; p = 0.039) (**Table 3**). As previously noted, this improvement did not have a significant impact on the 1-year and 2-year OS rates. Kaplan-Meyer curves were created to illustrate the OS and mortality rate for HGG in both cohorts (**Figure 1B**). Mortality rate at 1 and 2 years for LGG was 0 in both groups.

**Table 3:** Clinical outcomes and costs per intervention by resource use.

		Conventional OR	LF-iMR	LF-iMR- Conventional OR)	p-value
**Clinical outcomes**					
**Gross Total Resection (%)^a^**		56.2	70.0	13.8	0.121
**Complications (%)^b^**		21.3	14.0	−7.3	0.639
Postoperative KPS^*^		77 +/− 18	84 +/− 8	7	0.000
	Range	60–100	60–100		
**1-year OS (%)**		84.3	88	3.7	0.874
**2-years OS (%)**		73.3	70	−3.3	0.744
R-KPS (%)^c,*^		35	52	17	0.039
**1-year PFS (days)**		295	333	38	0.025

**Costs per intervention**					
**Operating Room**		3,356 €	5,162	1,806	0.0000
**ICU**		717 €	472 €	−245 €	0.0375
**Diagnostic images**		1,269 €	1,082 €	−186 €	0.0473
**Hospitalization**		5,087 €	4,177 €	−910 €	0.0332

ICU, Intensive Care Unit; KPS, Karnofsky performance status; OS, overall survival; PFS, Progression-free survival; R-KPS, Resection and postoperative KPS.

^*^Effectiveness measures considered in the cost-effectiveness analysis.

^a^Percentage of patients.

^b^Percentage of patients who suffered one or more complications.

^c^Percentage of patients presenting with a Subtotal or Gross total resection and an equal or higher postoperative KPS.

### Resource Use and Costs

**Figure 2:**
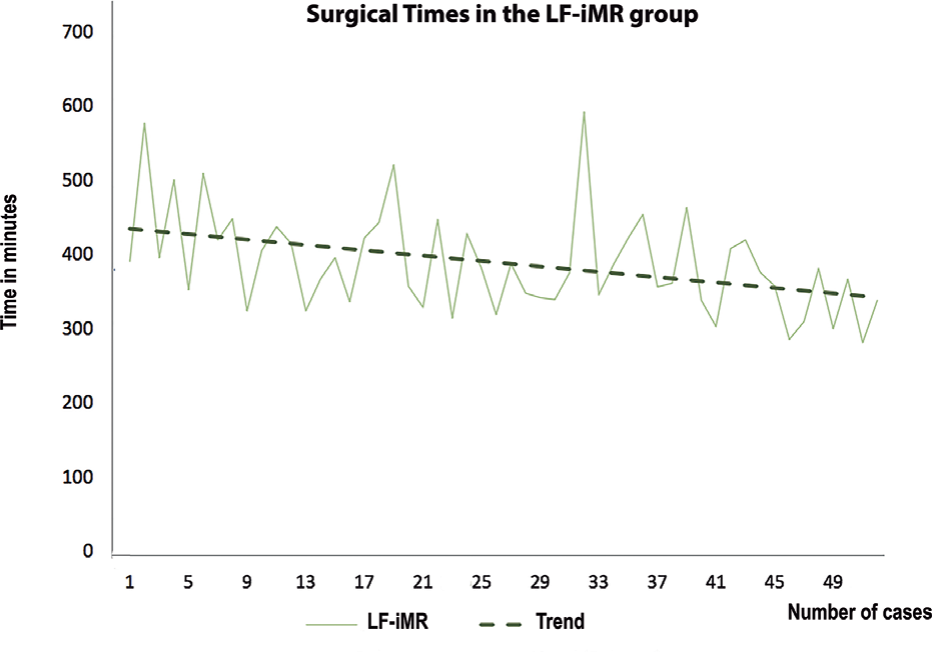
Surgical times per patient. The graphic illustrates the downward trend in surgical times progressively achieved in the LF-iMR group.

Regarding the use of resources, we stated a significant reduction in the LoS (9.7 vs. 11.9, p < 0.05) for patients in the LF-iMR group (**Table 1**). However, the cost per surgery was higher in the intervention group (+2,182 Euro). These costs are mainly due to the implementation of certain expendables and to longer surgical times. However, within the LF-iMR group there was a downward trend in surgical times as experience was progressively acquired which contributed to alleviate the overall cost due to operating room usage (**Figure 2**).

### Cost-Effectiveness

The results of the cost-effectiveness analysis showed a mean incremental cost per patient estimated in 789 € when the LF-iMR was used. Higher costs in the LF-iMR group were mainly due operating room usage; while ICU, diagnostic imaging and hospitalization costs were lower (**Table 3**). ICERs were 111 € per additional point of pKPS, 46 € per additional percentage point in the R-KPS and 21 € per additional day free of progression (**Table 4**). Therefore, the LF-iMR would be cost-effective depending on the willingness to pay of the hospital managers for an additional effectiveness unit. It is worth to note that the LoS is the cost variable that most affected the cost-effectiveness results.

**Table 4:** Results of the cost-effectiveness analysis.

		Conventional OR	LF-iMR	Incremental (iMR – Conventional OR)
**Cost**	**(€ 2018)**	11,599	10,810	789
**Effectiveness measures**	**Postoperative KPS**	77.1	84.3	7.1
	**R-KPS**	35	52	17
	**1-year PFS**	295	333	38
**ICER**	**Postoperative KPS**	n.a.	n.a.	111
	**R- KPS**	n.a.	n.a.	46
	**1-year PFS**	n.a.	n.a.	21

ICER, Incremental cost-effectiveness ratio: cost per additional unit of effectiveness (per additional point of postoperative KPS, percentage point of R-KPS or days of PFS); KPS, Karnofsky performance status; n.a., not-applicable; PFS, Progression-free survival; R-KPS, Resection and postoperative KPS.

**Figure 3:**
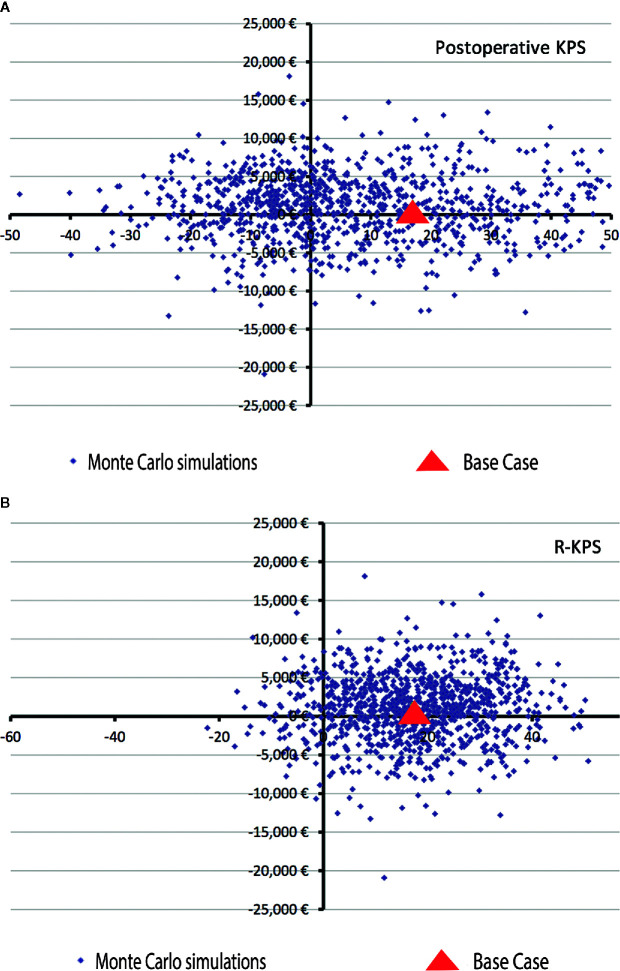
Monte Carlo simulations for the probabilistic sensitive analysis of cost-effectiveness results. **(A)** KPS. ICER for the postoperative KPS is horizontally dispersed on the plane, meaning that the results of the cost-effectiveness analysis are notably influenced by the values of this variable. **(B)** R-KPS. In the other hand, ICERs for R-KPS are largely located (35%) in the area where the iMR is a dominant technology, i.e., a less costly and more effective technology than the conventional OR. ICER, Incremental cost-effectiveness ratio; X-axis, Effectiveness measures; Y-axis, Cost in euros.

According to the deterministic sensitivity analysis, the iMR remains very close to a dominant position across the three different scenarios (**Table 5**). This means that the LF-iMR it is definitively more effective but slightly costlier than the conventional OR. Exceptionally, in the most conservative scenario A, where the clinical outcomes were set to be the worst possible results, the LF-iMR is dominated by the conventional OR in terms of KPS and 1-year PFS (i.e., costlier and less effective); however, the LF-iMR remains very close to the dominant position in terms of R-KPS. Regarding Scenario D, in which the analysis included exclusively patients diagnosed with HGG, displayed very similar results to the base case. PSA results are summarized and illustrated in **Figure 3**. The ICERs (Monte Carlo simulations) provided by the PSA for R-KPS as effectiveness measure, are largely located (35%) in the area (south-east quadrant of the plane) where the LF-iMR is a dominant technology (less costly and more effective).

**Table 5:** Deterministic sensitivity analysis results.

Parameter	Mean Value (SD)	ICER
Base Case		
LF-iMR device cost^a,b^	1,000,000 (200,000)	n.a.
Cost (€ 2018) (iMR)	11,599	
Cost (€ 2018) (Conv. OR)	10,810	
Postoperative KPS (iMR)^b^	84 (9)	111
Postoperative KPS (Conv. OR)^b^	77.1 (18)	
R-KPS ^b^	52 (10.4)	46
R-KPS (Conv. OR)^b^	35 (7)	
1-year PFS (iMR)^b^	333 (76)	21
1-year PFS (Conv. OR)^b^	295 (40)	
	**New Value on the Sensitivity Analysis**	
**Scenario A (conservative)^c^**		
Postoperative KPS (iMR)	75	Dominated
R-KPS	41.6	118
1-year PFS	255	Dominated
**Scenario B (favorable)^d^**		
Postoperative (iMR)	93	50
R-KPS	62.4	29
1-year PFS	410	7
**Scenario C (favorable)^e^**		
LF-iMR device cost	800,000^e^ 408^f^	n.a.
Postoperative KPS (iMR)	84	88
R-KPS	52	36
**Scenario D (HGG sub-group)^g^**		
Cost (€ 2018) (iMR)	11,608	n.a.
Cost (€ 2018) (Conv. OR)	10,853	
Postoperative KPS (iMR)	84.1	88
Postoperative KPS (Conv. OR)	75.5	
R-KPS (iMR)	55	44
R-KPS (Conv. OR)	31	
1-year PFS (iMR)	322	20
1-year PFS (Conv. OR)	284	

ICER, Incremental cost-effectiveness ratio: cost per additional unit of effectiveness (per additional point of postoperative KPS or percentage point of R-KPS); KPS, Karnofsky performance status; R-KPS, Resection and postoperative KPS; SD, Standard deviation.

^a^Total cost of the iMR device.

^b^SD is based on the variation of 20% as recommended in the international guidelines in health economic evaluation (18).

^c^Scenario A: Worst possible clinical outcomes of the iMR.

^d^Scenario B: Best possible clinical outcomes of the iMR.

^e^Scenario C: Reduction of the cost of the iMR device due to its extensive use.

^f^Cost per intervention.

^g^ Scenario D: Only considering the HGG cohort.

## Discussion

In this article, we have conducted a cost-effectiveness analysis of the impact of a LF-iMR in glioma surgery. The results demonstrated a significant improvement in the subset of patients operated with LF-iMR in terms of pKPS and in the number of patients in which a subtotal or GTR is achieved preserving their clinical status, but we failed to obtain statistically significant results regarding OS. The cost-effectiveness analysis demonstrated that the LF-iMR remained very close to a dominant position (i.e., a less costly and more effective technology than the conventional technique) for the analyzed effectiveness variable (R-KPS, postoperative KPS, and 1-year PFS). These benefits being acknowledged, whether the incremental costs are justified or not, might be matter of an open debate. Such a discussion involves socio-economic and ethical considerations that go beyond the purpose of the present article. Similar outcomes might lead to different decisions depending, for instance, on the willingness to pay of hospital managers for an additional effectiveness unit. Nevertheless, both the deterministic and PSA provided consistent results regarding the R-KPS.

Many groups have outlined the advantages of performing neurooncological surgery with the assistance of an iMR (13–15, 20). Moreover, it has been consistently reported a variable but clear upward trend in the EoR of gliomas (13, 15, 21–23). However, the vast majority of reports have failed to demonstrate a clinically relevant impact for patients (24, 25). Indeed, little has been said about the costs of these technologies, and even less about their cost-effectiveness and rationale for their implementation in public health systems (26). In previous publications, our group has highlighted the advantages and limitations of a low-field compared to a high-field iMR (13–15). Hence, in the current discussion, we will obviate differences regarding the novelty or technical specifications of the devices themselves. Moreover, future research in the evaluation of new technologies in neurosurgery may reproduce our methodology regardless of our results or the obsolescence of the technology. In this sense, it is important to note that commercial production of the device implemented in the present study ceased 1 year ago. This being said, we consider that our method is reproducible and that our results and conclusions could be useful to evaluate and compare other intraoperative imaging technologies implemented in neurooncological surgeries.

The available bibliography comparing conventional and LF-iMR guided surgery for gliomas is quite scarce (25). Senft et al. demonstrated in their randomized trial the augmentation in the rate of GTR in HGG without any impact in postoperative complications rates in the LF-iMR group (21). The EoR reported by these authors was superior to ours, 96% vs. 70% in the LF-iMR group and 68% vs. 56.2% in the conventional surgery group. It is worth to note that our cohorts included patients with LGG and HGG and that tumors near eloquent areas were not excluded. Therefore, an inferior EoR could be expected compared to those reports exclusively including contrast enhancing lesions in non-eloquent areas (21). The experience reported by Kubben et al. could hardly be extrapolated since their trial was stopped with only seven patients per branch due to slow recruitment, technical issues, and surgical time concerns (25, 27).

In this investigation, a significant decline in the LoS in the cohort operated with LF-iMR contributed to alleviate the incremental cost of the device itself. Various factors might accrue to explain this reduction. First, patient’s postoperative KPS was higher in the LF-iMR cohort, probably anticipating the date of discharge. Second, fewer complications resulted in a shorter ICU and in-bed stay. Finally, some of our patients are referred from other hospitals where preoperative MRIs does not include a study suitable for navigation. While this situation delayed the surgery in the cohort operated in a conventional fashion, it had no effects in the iMR cohort since their preoperative, not suitable for navigation, study could be merged with an immediately preoperative study acquired with the LF-iMR. Regarding LoS other authors had reported similar reductions, albeit they failed to achieve statistically significance (26).

Various reports have failed to demonstrate a significant improvement in OS in patients operated with the guidance of an intraoperative device despite higher GTR rates (13, 20, 25, 28). One-year analysis showed a slight decline in the mortality rate found in the intervention group (15.75% vs. 12%). Nonetheless, since this result was not statistically significant, we feared that it might be due to differences in the proportion of HGG and to short follow up. Therefore, follow up was extended to two years to check if this positive trend was sustained over time. Sadly, as previously reported, we could only confirm that small increases in GTR rates for HGG yields minor or none improvement in OS rates (**Figure 1**). Actually, OS rate at two years was lower in the intervention group when LGG and HGG were conjointly considered. This fact might seem inconsistent with the accepted statement of EoR being a major prognostic factor, it is clear that is not the only one. Probably, other prognostic factors such as the histological subtype, molecular biology or availability of therapeutic targets, and adjuvant therapies might play a more significant role (29–34).

In the present study, differences on PFS between both cohorts reached statistical significance. Our data are in line with previous reports in which conventional surgery and complete adjuvant therapy were implemented to achieve a mean PFS of approximately 9 months in HGG (35). Our PFS is superior to this figure in both cohorts probably due to the coexistence of HGG and LGG in each group. Previous reports evaluating the effectiveness of iMR devices failed to demonstrate significant changes on PFS (36). However, positive trends on the improvement of PFS had been linked to the use of intraoperative imaging tools (10, 36, 37) as a result of an increase in the EoR (35, 38, 39).

Regarding the selection of effectiveness variables, it is worth to note that 1-year PFS and KPS have been extensively employed and validated as adequate effectiveness variables. However, R-KPS, as a novel variable, has not previously been implemented in neuro-oncology studies and would require further validation. EoR was not considered as an effectiveness variable according to WHO recommendations which state that effectiveness should be evaluated by a variable that quantifies the gain of health (40). EoR is not an actual gain of health unlike KPS, PFS, or OS.

This clinical investigation harbors various limitations that should be disclosed. The hybrid design of the recruitment might involve a selection bias since patients were not randomly allocated to either group. In addition, both cohorts are made up of LGG and HGG, which confers a great clinical, radiological and prognostic heterogeneity that could be detrimental for the quality of the study. Actually, the range of diagnoses included in each cohort could independently affect the results reported. However, we sought to assess the effectiveness and implications of adding up a new device to the conventional surgical armamentarium. Therefore, we considered that the effects of surgery on LGG and HGG were comparable. Indeed, we limited our observations to 1 year, not to distort the effects of surgery with those owed to the intrinsic nature of tumors and adjuvant therapies. This decision limited the analysis of cost-effectiveness analysis regarding OS and PFS to 1 year since data of costs per patient were available just for one-year frame, which might be a short period, especially for LGG. Patients were operated by the same surgical team in both cohorts; therefore, differences in the clinical outcomes between groups should not be imputed to any improvement in the surgical technique. However, there may exist uncontrolled biases due to the retrospective collection of the historical cohort and unnoticed changes on surgical technique throughout the time frame of this study.

It might be argued that the design of the study downsizes the level of evidence of this clinical investigation. A prospective controlled trial would be the proper design to support with the highest level of evidence the conclusions herein stated (41). However, randomized controlled trials are often not adequate for neurological surgery since surgical trials are unmasked, surgical skills are not constant, lack of equipoise in decision makers, low-caseload diseases, slow recruitment, results in selection biases, and tremendous difficulties to obtain well-matched groups after randomization (42–44). In addition, the hybrid, retrospective and prospective, collection of patients did not allow the performance of a propensity score matching analysis. Thus, our approach sought to minimize biases while providing patients with the best possible treatment. At the moment of design of this research, the available bibliography on LF-iMR guided surgery was scarce; however, we considered it would not have been ethical to allocate patients randomly. Previous experience in intraoperative image guiding tools such as neuronavigation or CT scans suggested that LF-iMR could be useful, safe, and would result in similar or better surgical results compared to conventional surgery. Therefore, despite the limitations, our methodology responds to give patients the standard of care, to maintain an ethical perspective by providing a potentially beneficial treatment while achieving an acceptable level of evidence.

Regarding the cost-effectiveness analysis, patient perspective was indirectly considered by assessing clinical variables. However, since differences in patient complications and in 1-year survival were not statistically significant, these two variables were not included in the cost-effectiveness analysis. It might also be argued that Health-Related Quality of Life (HRQoL) should have been assessed before and after the intervention in order to provide effectiveness outcomes in terms of Quality-Adjusted Life Years (QALYs). This kind of measurements are often recommended to suggest more accurate and specific recommendations relying in country-specific cost-effectiveness thresholds (45).

## Conclusion

The present clinical research is, to the best of our knowledge, the first to provide evidence in terms of cost-effectiveness regarding LF-iMR implementation for glioma surgery. The guidance of an LF-iMR contributed to improve the clinical and surgical outcomes of the patients in which it was used. Despite the initially promising results in the LF-iMR cohort, they did not come along with significant higher survival rates. Therefore, we could conclude that patients operated under the guidance of a LF-iMR device achieve greater resections with less complications, better PFS rates but similar life expectancy. The LF-iMR is very close to be a dominant technology compared to conventional surgery in terms of R-KPS, pKPS, and 1-year PFS. These results are supported by the sensitivity analyses in terms of R-KPS, in which a dominant position of the LF-iMR seems largely plausible. Summarizing, in the light of our results, we recommend the use of LF-iMR as a cost-effective technology to achieve better outcomes in terms of R-KPS. However, for the rest of effectiveness, variables further research involving bigger cohorts and longer follow-up is required.

## Data Availability Statement

The original contributions presented in the study are included in the article/supplementary materials. Further inquiries can be directed to the corresponding author.

## Ethics Statement

The studies involving human participants were reviewed and approved by Comité Ético de Investigación Clínica Hospital Clinic de Barcelona HCB/2013/8782. The patients/participants provided their written informed consent to participate in this study.

## Author Contributions

SG-G, JG-S, LS-C, and JE conceived the presented idea. SG-G and BG-L led the present study, developed the theory, and performed the computations. SG-G, BG-L, PR, GR, and LS-C verified the analytical methods. Clinical data was collected and analyzed by SG-G, JG-S, DC, JT, and LR. Economical information was collected and interpreted by BG-L, GR, EA, IP, and LS-C. Figures and Tables were designed and created by SG-G, BG-L, and PR. All authors contributed to the article and approved the submitted version. SG-G and BG-L wrote the manuscript with support from PR, LS-C, and JE. SG-G edited the images and prepared them for submission. BG-L and SG-G checked the latest details of the manuscript and submitted it. EA, LS-C, and JE gave institutional, material, and logistic support. SG-G and BG-L revised the document considering the suggestions of the reviewers and submitted the final version of the manuscript.

## Conflict of Interest

The authors declare that the research was conducted in the absence of any commercial or financial relationships that could be construed as a potential conflict of interest.
